# High Cholesterol Diet Induces IL-1β Expression in Adult but Not Larval Zebrafish

**DOI:** 10.1371/journal.pone.0066970

**Published:** 2013-06-25

**Authors:** Yina Yoon, Jihye Yoon, Man-Young Jang, Yirang Na, Youngho Ko, Jae-Hoon Choi, Seung Hyeok Seok

**Affiliations:** 1 Department of Microbiology and Immunology, and Institute of Endemic Disease, College of Medicine, Seoul National University, Seoul, Republic of Korea; 2 Department of Life Science, College of Natural Sciences and Research Institute for Natural Sciences, Hanyang University, Seoul, Republic of Korea; National Institutes of Health, United States of America

## Abstract

Recently, it has been demonstrated that high cholesterol diet induced hypercholesterolemia and vascular lipid oxidation and accumulation in zebrafish larvae, suggesting that zebrafish is a new promising atherosclerosis model in addition to mouse models. However, up to date, there was no report regarding inflammatory cytokine expression during the lipid accumulation in zebrafish larva and adult fish. In this study, we first demonstrated the expression levels of IL-1β and TNF-α in high cholesterol diet (HCD)-fed zebrafish larvae, and found that although HCD induced vascular lipid accumulation, the cytokine expressions in the larvae were not changed by HCD. Furthermore, there was no significant difference in leukocyte accumulation in vessels between control and HCD fed group. But prolonged HCD induced IL-1β expression in spleen and liver compared to those of control zebrafish, and produced very early stage of fatty streak lesion in dorsal aorta of 19 week HCD-fed zebrafish. These results indicate that HCD induced hypercholesterolemia and atherosclerotic changes in zebrafish are very early stage, and suggest the necessity of the generation of mutant zebrafish having a disruption in a lipid metabolism-related gene leading to severe hypercholesterolemia and advanced atherosclerosis.

## Introduction

Atherosclerosis is a chronic multifactoral inflammatory disease involving elastic and muscular arteries [1], [2]. The inflammatory response of the vascular wall is thought to involve uncontrolled proliferation of vascular myointimal cells, production of foam cells, and progressive vascular occlusion. The inflammatory cells in atherosclerotic lesions produce various cytokines including IL-1β, TNF-α, IL-6, MCP-1 so on, and these complex cytokine networks contribute to formation of atherosclerotic lesion [3].

The atherosclerotic processes in human continue for long time with high complexity. Furthermore, it is almost impossible to perform direct analysis of the atherosclerotic lesion from an individual patient. Therefore, several animal models including mouse, rat, rabbit, and pig have been used to understand the complicated processes of atherosclerosis [4]. Especially, mouse models have been widely used for understanding of atherosclerosis, and instrumental in evaluating new atherosclerotic drugs. Since wild type mice are highly resistant to atherosclerosis due to the high levels of antiatherosclerotic HDL, several transgenic and knockout mouse models were developed to increase the susceptibility to atherosclerosis [5]. The most widely used mouse models for atherosclerosis are apolipoprotein E–deficient and LDL receptor–deficient mice, and have contributed to understand the mechanisms of atherogenesis.

In addition to mouse model, Miler and colleagues recently developed a zebrafish model of hyperlipidemia and atherosclerosis [6]. Zebrafish has been widely used, especially in developmental biology and neuroscience, because many developmental and physiological processes are similar to those of mammals [7]. Other advantages of zebrafish are relatively low cost for maintenance, rapid development and ease of genetic manipulation [8]. In particular, an important advantage of zebrafishes is transparent during the larva stage so that it is possible to observe internal organs including heart, blood, and vessel so on in live fish [9]. Therefore, larvae are highly useful in vascular research. The transgenic *fli1*:EGFP zebrafish, which express enhanced green fluorescent protein (GFP) in the vascular endothelium, is very useful to visualize the whole vascular system in live animals, and widely used to analyze angiogenesis [10]. Recently, Miler group fed zebrafish with high cholesterol diet, and found the accumulations of lipids and myeloid cells in vessel wall [6]. Furthermore, they successfully evaluated the validity of antibody against oxidized epitopes as an anti-atherogenic agent using zebrafish larvae model [11]. Thus, it is likely that zebrafish will obtain popularity in atherosclerosis study. However, before getting more popularity as atherosclerosis model, detailed analysis on the atherosclerotic changes by high cholesterol diet is required in zebrafish model.

To date, although high cholesterol diet induced lipid and myeloid cell accumulations in larva and adult zebrafish, there was no report on the expression profiles of inflammatory cytokines in these fishes. In this study, we analyzed the expressions of inflammatory cytokines in zebrafish larva and in adult zebrafish fed high cholesterol diet. Especially, we focused on the analysis of the expression of IL-1β and TNF-α, mainly produced during innate immune response induced by high cholesterol diet in larvae. And then, we also continued to see the cytokine expressions in adult zebrafish fed same diet for longer period.

## Materials and Methods

### Zebrafish Maintenance and Feeding

All animal studies were approved by the Animal Care and Use Committee of the Seoul National University (SNU-120102-7). *Fli*:EGFP transgenic zebrafish (*Danio rerio*) was from Zebrafish Oraganogenesis Mutant Bank (ZOMB) in Korea. *Cd45*:DsRed transgenic zebrafish was a generous gift of Dr. David Traver from university of California, San Diego. Zebrafish embryos were obtained from natural spawning and grown in tank water at 28°C on a 14-hour light, 10-hour-dark cycle. A high-cholesterol diet (HCD) for zebrafish was made in a diethyl ether solution of cholesterol (Sigma) to achieve a content of 4% (wt/wt) cholesterol in the artificial artemia (Azoo) after ether evaporation. Zebrafish was fed the diet 3 times a day for 19 weeks. To visualize vascular lipid accumulation in larvae, both control and HCD food were supplemented with 10 µg/g of a fluorescent cholesteryl ester analog (cholesteryl BODIPY 576/589-C11) (Invitrogen).

### Measurement of Total Cholesterol

More than two microliters of blood was extracted from the hearts of zebrafish of control and HCD groups, after HCD feeding for 19 weeks. PBS-EDTA(final concentration, 1 mM) was added and plasma extracted from 10 zebrafish from each group was obtained by centrifugation. Total Cholesterol was determined using commercial assay kits (Asan Pharm., Seoul, Korea).

### RNA Isolation and Reverse Transcription PCR

At 8, 10, 15 and 20 days after control and HCD feeding from 4 day post fertilization (dpf), larvae (n = 15∼20) were pooled with same concentration. Those larvae were homogenized in 1.0 ml Trizol reagent (Bioneer, Daejeon, Korea), and total RNA was extracted from the homogenization, according to the manufacturer’s protocol. RNA was used for cDNA synthesis with SuperScript3 Reverse Transcriptase kit (Invitrogen). Total RNA from spleen and liver from 19 week old zebrafish were extracted using Trizol reagent. 20 larvae at 48 hour post fertilization (hpf) were stimulated with LPS from *Salmonella typhimurium* (Sigma) at 50 µg/ml for 3 hours, then washed in sterilized water for positive control of pro-inflammatory cytokines expression. The sequences of PCR primers for messenger RNA detection were as followings; 5′-GAGGAGCACCCCGTCCTGCTCAC-3′ and 5′-GATGGCTGGACCAGGGCCTCTGG-3′ for beta actin (Genbank: AF057040), 5′-TGGCGA ACGTCATCCAAG-3′ and 5′-GGAGCACTGGGCGACGCATA-3′ for IL-1β (Genbank: AY340959), and 5′-GCTTATGAGCCATGCAGTGA-3′ and 5′-TGCCCAGTCTGTCTCCTTCT-3′ for TNF-α (GenBank: AY427649). PCR was performed with an initial denaturation of 5 minutes at 95°C, then 35 cycles were run for 30 seconds of denaturation at 95°C, 30 seconds of annealing at 55°C, and 30 seconds of extension at 72°C. The reacted products were electrophoresed on a 1.5% agarose gel. Quantitative real-time PCR (SYBRgreen) assays were performed using a Rotor-Gene Q (QIAGEN) with zebrafish IL-1β, TNF-α and IL-6. The primer sequences of IL-1β and TNF-α are same to the primers used in semiquantitative RT-PCR. The sequence of PCR primer for IL-6 mRNA detection is as following; 5′-TCAACTTCTCCAGCGTGATG-3′ 5′-TCTTTCCCTCTTTTCCTCCT G-3′.

### *In vivo* Confocal Microscopy

For in vivo confocal microscopy, anesthetized fish larvae were housed in a sealed, temperature controlled room in a small drop of tricaine containing water. Zebrafish larvae were examined with an Olympus FV1000 confocal laser scanning microscope (Olympus, Tokyo, Japan). Images of zebrafish larvae were captured every 200 nm, and all images were analyzed and processed using the Olympus Fluoview software (Olympus).

### Histology

Zebrafish was anesthetized by putting them in low-temperature, and then cut off a head of each samples to exsanguinate. Spleens of zebrafish fed a HCD or control diet after 19 week feeding were weighed by (a×b^2^)/2, a = long length, b = short length [12]. Fishes were immersed in 4% paraformaldehyde for a day or two days at room temperature. Next, at the end of fixation, samples were decalcified by decalcification buffer containing the 8% Hydrochloric Acid and 8% Formic Acid for 3 days. Then samples for paraffin section were dehydrated by tissue processing and embedded in paraffin. In case of cryosection, samples were immersed in 30% sucrose for dehydration, and were embedded in OCT compound. The blocks were sectioned at 5-µm for staining with hematoxylin and eosin or Oil-Red O. For lipid staining, the sections were stained with oil red O for 4 hours, washed with PBS briefly. The stained sections were digitally photographed.

### Statistical Analysis

Two-tailed parametric Student t-test or non-parametric Mann Whitney analysis were used for statistical analyses (*P<0.05).

## Results

### Vascular Lipid Accumulation in HCD-fed Zebrafish Larvae

**Figure 1:**
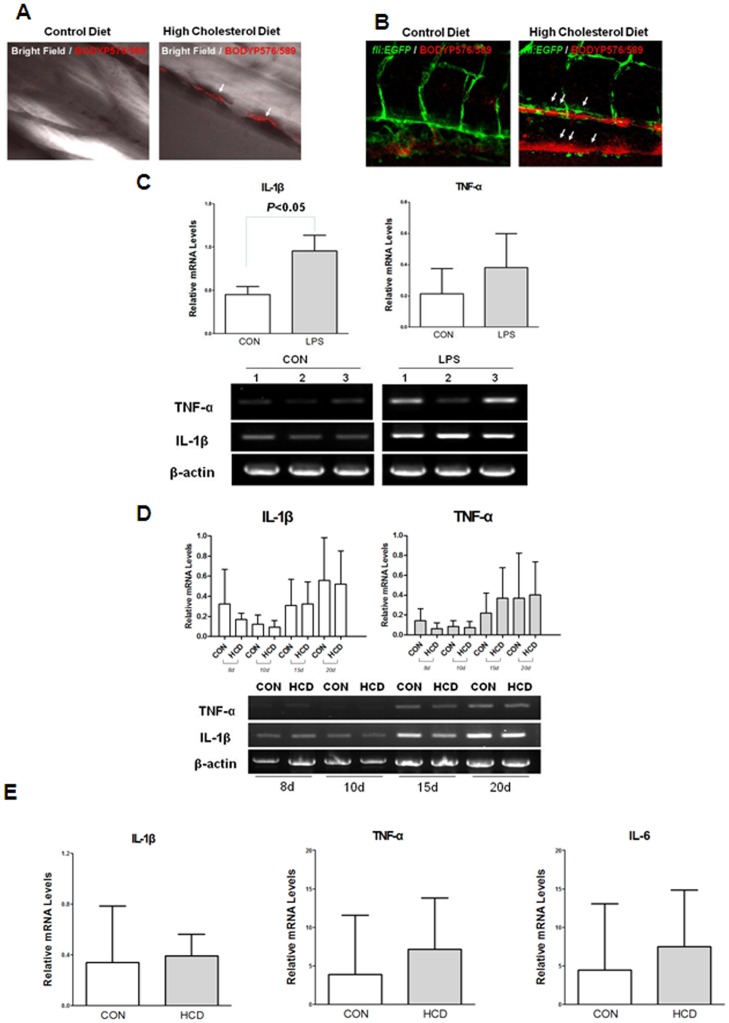
Lipid accumulation and pro-inflammatory cytokine level in zebrafish larvae. A and B, Five-day old wild (A) and *fli1:EGFP* (B) zebrafish larvae were fed a 4% cholesterol-enriched (HCD) or a normal (control) diet for 10 days, both supplemented with a 10 µg/g red fluorescent lipid. Images of the caudal vasculature in live larvae show vascular lipid accumulation visualized by lipid-associated BODIPY 576/589 fluorophore deposits in HCD fed larvae. The larger lipid deposits were found at the bifurcation site (arrows). Scale = 40 µm. C. Increased expressions of IL-1β and TNF-α by 3 h LPS treatment at 2-day old zebrafish larvae. Semi-quantitative PCR was performed for IL-1β and TNF-α in triplicate (n = 20 zebrafish larvae per group) D. Semi-quantitative PCR for IL-1β and TNF-α expressions from zebrafish larvae fed a HCD enriched with 4% cholesterol or normal (control) diet for 8 days to 20 days. The experiment was performed in triplicate (n = 15∼20 zebrafish larvae per group) E. Realtime RT-PCR analysis for the expression levels of IL-1β, TNF-α and IL-6 from zebrafish larvae fed a HCD enriched with 4% cholesterol or normal (control) diet for 10 days. Results are representative for at least three independent assays (n = 15–20 zebrafish larvae per one experiment).

To detect the lipid accumulation in HCD-fed zebrafish larvae, five-day-old wild and *fli1:EGFP* zebrafish larvae were fed HCD or control diet for 10 days, both supplemented with a 10 µg/g red fluorescent lipid. We observed that caudal vasculature of live anesthetized zebrafish larvae (Figure 1). Remarkably, only in HCD-fed larvae, there were focal areas of bright red fluorescence in blood vessels, which were interpreted as lipid accumulation in the vessel wall (Figure 1A). Although some deposits were found in the dorsal aorta and at the sites of blood vessel bifurcation as well, the majority of lipid deposits were observed in the caudal vein (Figure 1B).

### Pro-inflammatory Cytokine Level in HCD-fed Zebrafish Larvae

We explored whether HCD leads to increase pro-inflammatory cytokine level in zebrafish larvae. First, to assess the ability of zebrafish larve to express cytokines, Salmonella LPS were treated to 2-day old zebrafish larvae, and induced the 2-fold higher of IL-1β expression compared to untreated control (Figure 1C). Also TNF-α was observed the increased pattern in LPS treated zebrafish larvae compared with control diet. Thus we started the HCD feeding and continued it for 20 days. Statistically no differences of the expression level of IL-1β and TNF-α were observed between zebrafish larvae fed a HCD enriched with 4% cholesterol group and a control diet (Figure 1D). To confirm the cytokine expressions in high cholesterol fed larvae, we performed realtime RT-PCR for the expressions of IL-1β, TNF-α and IL-6, and found that there are no differences in the expression levels of these cytokines between groups (Figure 1E). These results suggest that pro-inflammatory cytokine levels did not be changed at zebrafish larvae stages with a HCD.

### Vascular Leukocyte Accumulation in HCD-fed Zebrafish Larvae

**Figure 2:**
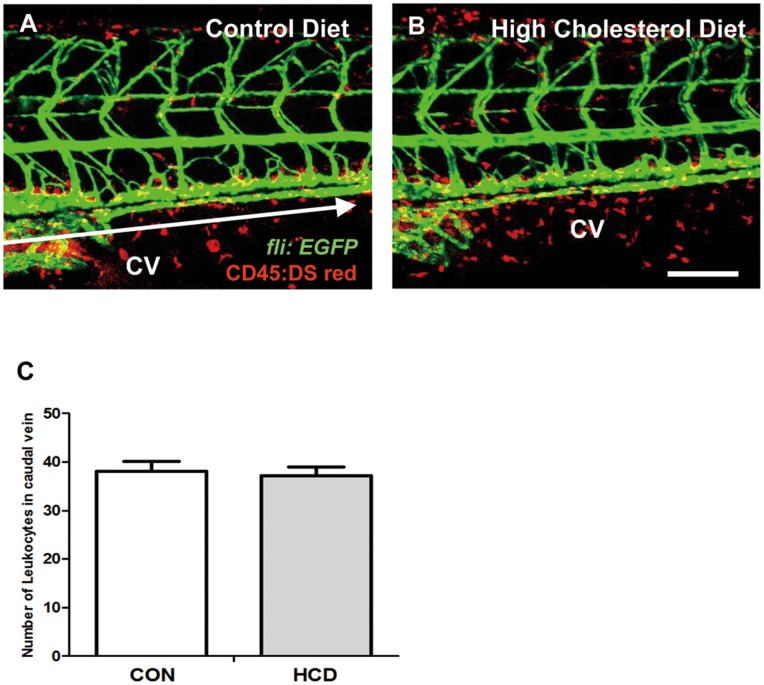
Vascular leukocytes accumulation in HCD fed zebrafish. zebrafish larvae were fed a normal (control) diet (A) or a 4% cholesterol-enriched (HCD) (B) for 10 days. Images of the caudal vasculature in live larvae show leukocytes accumulation in blood vessel or around tissues deposits in zebrafish larvae. Scale bar = 80 µm. The number of leukocytes in caudal vein (yellow) between each edge of arrow were counted in each a control diet and HCD group (each group n = 9) (C). CV, caudal vein.

To detect the leukocyte accumulation in HCD-fed zebrafish larvae, double transgenic (*fli*:EGFP; *Cd45*:DsRed) larvae were fed HCD or control diet for 10 days. We observed that caudal vasculature of live anesthetized zebrafish larvae from control and HCD groups (Figure 2A, 2B). But there was no significant difference in vascular leukocyte accumulation between control and HCD groups (Figure 2C).

### Hypercholesterolemia in HCD-fed Adult Zebrafish

**Figure 3:**
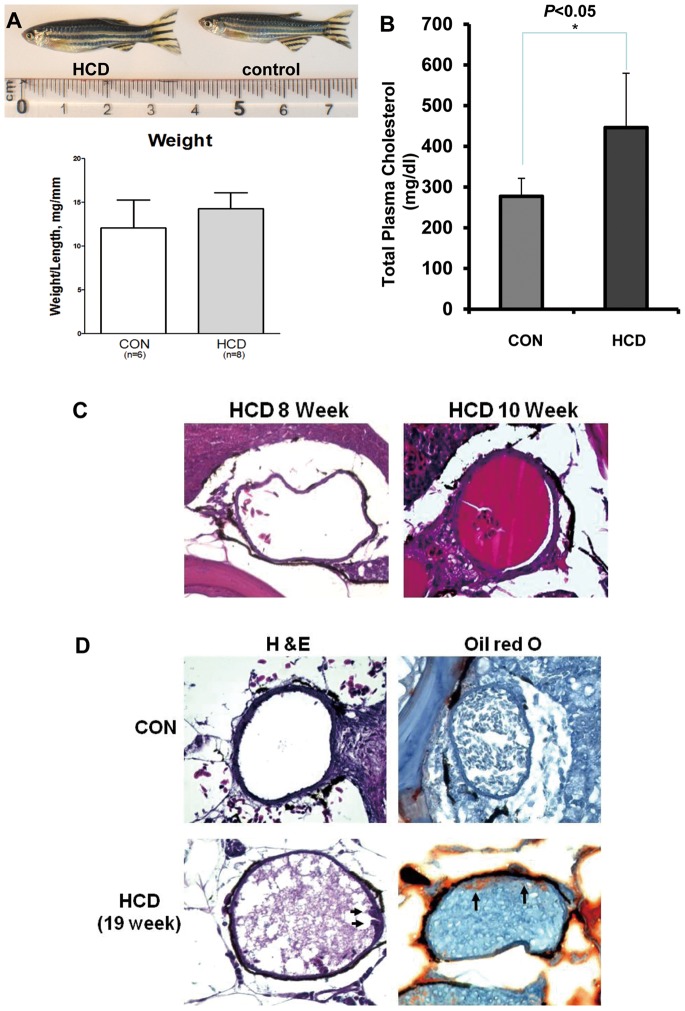
HCD-induced hypercholesterolemia and vascular lipid accumulation in adult zebrafish. Five-week-old zebrafish were fed a 4% cholesterol-enriched (HCD) or a normal diet (control) diet for 8, 10, and 19 weeks. A. Male fish (confirmed by dissection) fed a HCD or a control diet. The ratio of body weight to length (body mass index) (n = 6–8 in each group, males; no statistically significant differences). B. Total plasma cholesterol levels of zebrafish fed a HCD or a control diet after feeding 19 weeks (n = 10 in each group, both males and females). C and D. Dorsal aortas of HCD-fed zebrafish or control diet. HCD-fed zebrafish for 8 to 10 weeks; (C) H & E staining. (D) Fatty streak lesion formed in the doral aorta of 19 week HCD-fed zebrafish. Arrows show the lipid accumulation by Oil-Red O staining.

To test whether zebrafish are inherently susceptible to atherosclerosis induced by high cholesterol feeding, HCD was fed to zebrafish starting at 5 weeks post-fertilization (adult fish) for an additional 8 to 19 weeks. Compared to a control diet-fed zebrafish, a HCD-fed zebrafish had an enlarged belly, but the weight gain was not statistically different (Figure 3A). However, there was a significant increase in plasma total cholesterol level in HCD-fed zebrafish (Figure 3B). Dorsal aortas of a HCD-fed zebrafish for 8 to 10 weeks did not show any different changes compared to a control diet-fed zebrafish (Figure 3C). However, zebrafishes fed HCD for 19 weeks developed lipid accumulations in the wall of dorsal aorta, which can be seen by Oil-red O staining (Figure 3D). Such lesions were classified as very early stage of fatty streak lesion.

### Pro-inflammatory Cytokine Levels in HCD-fed Adult Zebrafish

**Figure 4:**
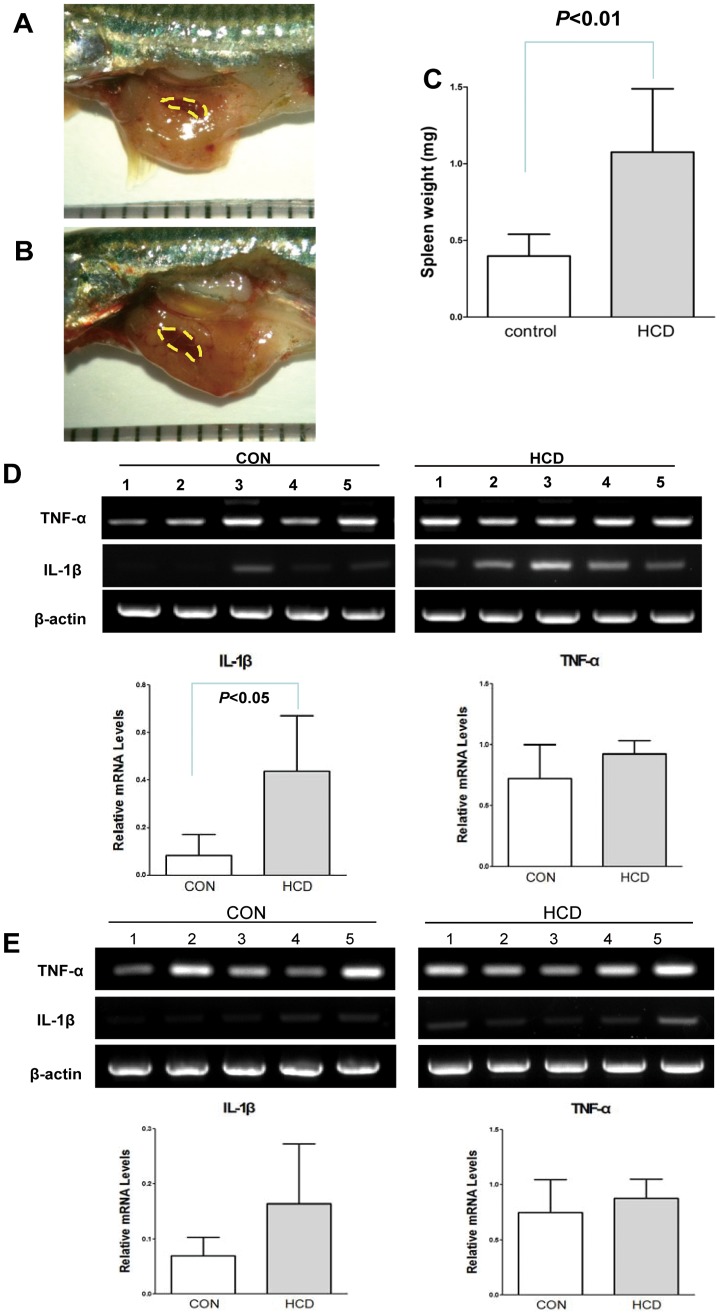
The expression levels of IL-1β and TNF-α in spleen from zebrafish fed HCD for 19 weeks. A and B, The size of spleen of zebrafish fed a normal diet (A, control) and a high-cholesterol diet (B, HCD) for 19 weeks. Each scale = 1 mm. C. Spleen weight of zebrafish fed a HCD or a control diet after 19 week feeding; Weight formula = (a×b^2^)/2, a = long length, b = short length. (n = 10 in each group, both males and females). D. The expression levels of IL-1β and TNF-α of the spleen from zebrafish fed a HCD or a control diet for 19 weeks. E. The expression levels of IL-1β and TNF-α of the liver from zebrafish fed a HCD or a control diet for 19 weeks. Semi-quantification PCR was performed for IL-1β and TNF-α. Results are representative for at least three independent assays (n = 5 in each group, males).

Feeding HCD to zebrafish, in which spleen enlargement was observed starting at 19 weeks (Figure 4A and B). Spleen weight of a HCD-fed zebrafish, compared to a control diet-fed zebrafish after 19 week feeding showed significant increase (Figure 4C). Upon spleen enlargement on HCD-fed zebrafish, we also confirmed the pro-inflammatory cytokine levels, IL-1β and TNF-α on spleen sample of a HCD-fed zebrafish. Semi-quantitative RT-PCR was performed for the evaluation of IL-1β and TNF-α expressions. IL-1β after 19 weeks feeding of HCD showed the 4-fold higher in HCD-fed zebrafish spleen compared with the control diet-fed zebrafish spleen (Figure 4D, *P*<0.05). However, the mRNA levels of TNF-α (Figure 4D) and IL-6 (data not shown) was observed no statistically differences between groups. In mouse model, high cholesterol diet has been known to induce hepatic fatty changes leading to hepatic inflammation [13]. To demonstrate that there was any change on liver of HCD-fed zebrafish, we examined the liver histology between a HCD-fed and a control diet-fed zebrafish group, and found that there were no differences in liver histology (data not shown). However, when we checked the pro-inflammatory cytokine levels, IL-1β and TNF-α on liver samples of a HCD-fed zebrafish or a control diet-fed zebrafish by semi-quantification PCR, we also found that IL-1β showed the increased pattern on HCD-fed zebrafish liver (*P* = 0.0603, Figure 4E).

## Discussion

Recently, HCD-fed zebrafish has been shown to be a promising animal model of atherosclerosis [6]. Especially, the important advantage of HCD-fed zebrafish model seems that the accumulation and oxidation of lipid can be visualized directly from the larva stage of zebrafish, which allows doing a time-course experiment. Furthermore, it has also been demonstrated that the treatment of oxidized epitope-specific antibody effectively prevented HCD-induced lipid accumulation in zebrafish larva [11].

According to the previous studies using human cells and mouse models, it is widely-accepted that retained lipoproteins in subintimal space can be oxidatively modified and the oxidized lipoproteins can induce the production of proinflammatory cytokines in endothelial cells, smooth muscle cells and monocytes leading to initiation of atherosclerosis [14]–[16]. Interleukins are key mediators of the systemic inflammatory response, and especially, IL-1β has been known to play an important role in atherosclerosis [17]. In atherosclerotic lesion, monocytes and macrophages, having key roles in atherosclerotic plaque biology, mainly produces IL-1β. The increased level of IL-1β has been shown to be related to rupture-prone atherosclerotic lesion [18], [19]. The IL-1β production can be stimulated by cholesterol crystal and modified LDL [20], [21]. The secretion of TNF-α also can be stimulated by oxidized LDL via activation of AP-1 [22]. The secreted TNF-α promotes cholesterol uptake and foam cell formation through NF-κB activation [23]. Thus TNF-α initiates a positive feedback leading to increased LDL uptake and consequent activation of TNF-α release. Since there was no clear study regarding the expressions of proinflammatory cytokines in HCD-fed zebrafish, we attempted to observe the IL-1β and TNF-α expressions to determine the involvement of inflammatory cytokines in the vascular lipid accumulation in HCD-fed zebrafish. In this study, we first confirmed that zebrafish larvae were stimulated by LPS treatment and produced IL-1β and TNF-α. These cytokines were also strongly induced by LPS stimulation in adult zebrafish (data not shown). We next observed the vascular lipid accumulation in HCD-fed larvae. However, although HCD significantly induced the lipid accumulation in the vasculature, IL-1β and TNF-α were not induced by HCD during the larva stage. Considering that, in previous studies, HCD induced inflammatory changes including leukocyte accumulation in vascular wall [6], [11], our results are somewhat unexpected. But the prolonged HCD for 19 weeks induced IL-1β expression. These results indicate that early lipid accumulation shown in larvae may not be highly related to the inflammatory processes. In addition, in human and mouse, the atherosclerotic inflammatory processes are occurred in arteries not in vein. However, the lipid accumulation is mainly occurred in the caudal vein of zebrafish larva, not in aorta. Thus, the early lipid accumulation in vessel of larvae may be somewhat different from the early atherosclerotic processes of mammalian system. Thus it needs further studies to determine the detailed processes of vascular lipid accumulation in HCD-fed zebrafish larvae.

The mice are quite resistant to atherosclerosis because of their high blood levels of anti-atherogenic HDL and low levels of proatherogenic LDL and VLDL [5]. Although C57BL/6 mice are susceptible mouse strain to atherosclerosis, they develop only very early stage of atherosclerotic lesion after 1.25% cholesterol diet for 34 weeks (unpublished data). Thus, mouse atherosclerotic models depend on generating high blood levels of LDL and VLDL induced by genetic ablations of LDL receptor (LDLR) and apolipoprotein E (ApoE). Likewise, zebrafish has been known to have high level of HDL, which is not very suitable system to study atherosclerosis [24]. In this study, we fed zebrafishes with HCD for 19 weeks. The total cholesterol levels were increased by only 60% in HDC fed zebrafish compared to control fish. And the current limitation of HCD-fed zebrafishes as atherosclerosis model is that they developed very early stage of fatty steak lesion. Therefore, to get better understanding about atherosclerotic processes and advanced atherosclerosis, the genetic ablation to remove lipid metabolism-related genes may induce severe hypercholesterolemia and advanced atherosclerosis in zebrafish. Recent advances in genetic manipulation allow inducing inheritable targeted gene inactivation in zebrafish. The targeted gene inactivation using zinc finger nuclease (ZFN) was performed by two groups. They successfully induced inheritable gene-targeted mutagenesis in zebrafish [25], [26]. Moreover, Transcription activator-like effector nucleases (TALENs) has been also adopted to induce targeted disruption of genes in zebrafish [27], [28].

These technologies are highly valuable to understand the function of certain gene in zebrafish. LDLR and ApoE, known as important molecules in mammalian lipid metabolism, are also conserved in zebrafish. Moreover, several lipid metabolism-related genes are conserved in zebrafish including ApoA1, ABCA1, and oxysterol-binding proteins so on [24]. Therefore, it seems that ZFN or TALEN-induced targeted mutagenesis on these genes may induce hypercholesterolemia or proatherogenic cascades in zebrafish leading to the generation of valuable model for advanced atherosclerosis.

In conclusion, although HCD effectively induced vascular lipid accumulation, the expressions of IL-1β and TNF-α were not increased in HCD-fed larvae. The prolonged HCD for 19 weeks induced IL-1β expression, but only produced very early fatty streak lesion in dorsal aorta. These results indicate that HCD induced hypercholesterolemia and atherosclerotic changes in zebrafish are very early stage, and suggest the necessity of the generation of mutant zebrafish having a disruption in a lipid metabolism-related gene leading to severe hypercholesterolemia and advanced atherosclerosis.
